# Antibacterial Activities of Metabolites from *Platanus occidentalis* (American sycamore) against Fish Pathogenic Bacteria

**DOI:** 10.4172/2155-9546.1000364

**Published:** 2015-10-15

**Authors:** Kevin K Schrader, Mark T Hamann, James D McChesney, Douglas L Rodenburg, Mohamed A Ibrahim

**Affiliations:** 1 United States Department of Agriculture, Agricultural Research Service, Natural Products Utilization Research Unit, National Center for Natural Products Research, Post Office Box 1848, Mississippi 38677, USA; 2 Departments of Pharmacognosy, School of Pharmacy, University of Mississippi, University, Mississippi 38677, USA; 3 Pharmacology, Chemistry and Biochemistry Department, School of Pharmacy, University of Mississippi, University, Mississippi 38677, USA; 4 Ironstone Separations, Inc., 147 County Road 245, Etta, Mississippi 38627, USA; 5 Department of Chemistry of Natural Compounds, National Research Center, Dokki 12622, Cairo, Egypt

**Keywords:** Antibacterial, Channel catfish, Columnaris disease, *Flavobacterium columnare*, Kaempferol, platanoside, Streptococcosis, *Streptococcus iniae*, Sycamore

## Abstract

One approach to the management of common fish diseases in aquaculture is the use of antibiotic-laden feed. However, there are public concerns about the use of antibiotics in agriculture and the potential development of antibiotic resistant bacteria. Therefore, the discovery of other environmentally safe natural compounds as alternatives to antibiotics would benefit the aquaculture industries. Four natural compounds, commonly called platanosides, [kaempferol 3-*O*-*α*-L-(2*″*,3*″*-di-*E*-*p*-coumaroyl)rhamnoside (**1**), kaempferol 3-*O*-*α*-L-(2*″*-*E*-*p*-coumaroyl-3*″*-*Z*-*p*-coumaroyl)rhamnoside (**2**), kaempferol 3-*O*-*α*-L-(2*″*-*Z*-*p*-coumaroyl-3*″*-*E*-*p*-coumaroyl)rhamnoside (**3**), and kaempferol 3-*O*-*α*-L-(2*″*,3*″*-di-*Z*-*p*-coumaroyl)rhamnoside (**4**)] isolated from the leaves of the American sycamore *(Platanus occidentalis*) tree were evaluated using a rapid bioassay for their antibacterial activities against common fish pathogenic bacteria including *Flavobacterium columnare*, *Edwardsiella ictaluri*, *Aeromonas hydrophila*, and *Streptococcus iniae*. The four isomers and a mixture of all four isomers were strongly antibacterial against isolates of *F. columnare* and *S. iniae*. Against *F. columnare* ALM-00-173, **3** and **4** showed the strongest antibacterial activities, with 24-h 50% inhibition concentration (IC_50_) values of 2.13 ± 0.11 and 2.62 ± 0.23 mg/L, respectively. Against *S. iniae* LA94-426, **4** had the strongest antibacterial activity, with 24-h IC_50_ of 1.87 ± 0.23 mg/L. Neither a mixture of the isomers nor any of the individual isomers were antibacterial against isolates of *E. ictaluri* and *A. hydrophila* at the test concentrations used in the study. Several of the isomers appear promising for the potential management of columnaris disease and streptococcosis in fish.

## Introduction

Common bacterial diseases of pond-raised channel catfish (*Ictalurus punctatus*) in the southeastern United States include columnaris disease and enteric septicemia of catfish (ESC). Columnaris disease-related problems also occur world-wide in many other species of freshwater fish (e.g., rainbow trout, salmon, and tilapia), and this disease can result in heavy economic losses to these aquaculture industries [1,2]. The etiological agent for columnaris disease is the bacterium *Flavobacterium columnare* which is a Gram-negative motile rod (2–10 μm in length) in the family Flavobacteriaceae [3]. Columnaris disease can result in severe necrosis of gill tissue, skin ulceration from systemic infection, and high mortalities in fish. The etiological agent for ESC is the bacterium *Edwardsiella ictaluri* which is a Gram-negative weakly motile rod (1–3 μm in length) and in the family Enterobacteriaceae [2]. The gross lesions of ESC in channel catfish can include pale gills, small depigmented lesions and/or ulcers (1–3 mm) on the backs of infected fish, open lesions along the central skull line between the eyes, and hemorrhage at the base of the fins, under the jaw, and on the belly. High mortality rates of channel catfish and prevention and treatment approaches can cost producers millions of dollars annually [2].

Bacterial diseases also adversely impact the aquaculture of sunshine bass, *Morone chrysops* female × *Morone saxatilis* male. Sunshine bass production has been the fastest growing segment of the aquaculture industry in the United States over the past decade and has spread to several countries in Europe and Asia [4,5]. However, sunshine bass production has been plagued by many pathogens including *Aeromonas hydrophila* and *F. columnare* [2]. *A. hydrophila* is a Gram-negative short, motile rod, ubiquitous in the aquatic environment, and the agent responsible for motile *Aeromonas* septicemia (MAS) in fish. Both *A. hydrophila* and *F. columnare* typically act as secondary pathogens causing significant losses in fish populations in the presence of predisposing stressors. These two pathogens can also act as primary pathogens, requiring no predisposing conditions to inflict significant mortalities [2].

Another bacterial disease which can cause losses of farmed freshwater fish is streptococcosis which is a prevalent problem in fish species such as tilapias (*Oreochromis* spp.) and hybrid striped bass [*Morone chrysops* female × *Morone saxatilis* male (Percichthyidae)] [6]. One of several streptococcal bacteria that has been attributed as the cause of streptococcosis in freshwater fish is *Streptococcus iniae* which is a Gram-positive spherical-shaped cell (0.5–2.0 μm in diameter). In tilapia, gross lesions of streptococcosis include hemorrhaging in the skin and at the base of fins, epidermal lesions and/or bloody ulcers, and opaque corneas [2]. Streptococcosis in a fish population can result in very high mortality rates, and therefore management approaches include prevention as well as treatment once the disease is determined to be present.

Catfish producers may use several currently available management approaches for columnaris disease or ESC including the application of medicated feeds [oxytetracycline dihydrate (Terramycin®; Phibro Animal Health, Teaneck, NJ, USA) for ESC and florfenicol (Aquaflor®; Intervet Inc., Millsboro, DE, USA) for columnaris disease and ESC], live attenuated vaccines [7], and nonantibiotic therapeutants such as 35% Perox-Aid^®^ for external columnaris [2]. However, Perox-Aid^®^ is not recommended for use in earthen ponds without water exchange. Additional inorganic agents such as copper sulfate pentahydrate (CuSO_4_.5H_2_O) and potassium permanganate (KMnO_4_) have been cited as potential treatments for columnaris disease [8], but the efficacy of these compounds can be adversely impacted by some water quality variables. Also, extra care must be used when utilizing these therapeutants due to their broad-spectrum toxicity towards non-target organisms such as channel catfish [9].

At present, there are no approved drugs for the treatment of MAS caused by *A. hydrophila* in sunshine bass. Recent studies examining the use of CuSO_4_.5H_2_O, KMnO_4_, and Aquaflor® have noted the potential efficacy of using these compounds to manage MAS in fish [10,11]. The use of Aquaflor® with CuSO_4_.5H_2_O to manage mixed infections of *A. hydrophila* and *F. columnare* in sunshine bass appears to be especially promising [10], but future studies in ponds are still required to confirm this approach.

Preventive management approaches for streptococcosis include maintaining high water quality, the application of high-quality diets, adequate water exchange and disinfection, and the removal of fecal waste from recirculating water systems [2]. Currently, only florfenicol (Aquaflor®) is approved in the United States for the treatment of streptococcal septicemia caused by *S. iniae* in warmwater fish. Vaccination appears to be a promising approach for protection against *S. iniae* infection in Nile tilapia [12].

**Figure 1:**
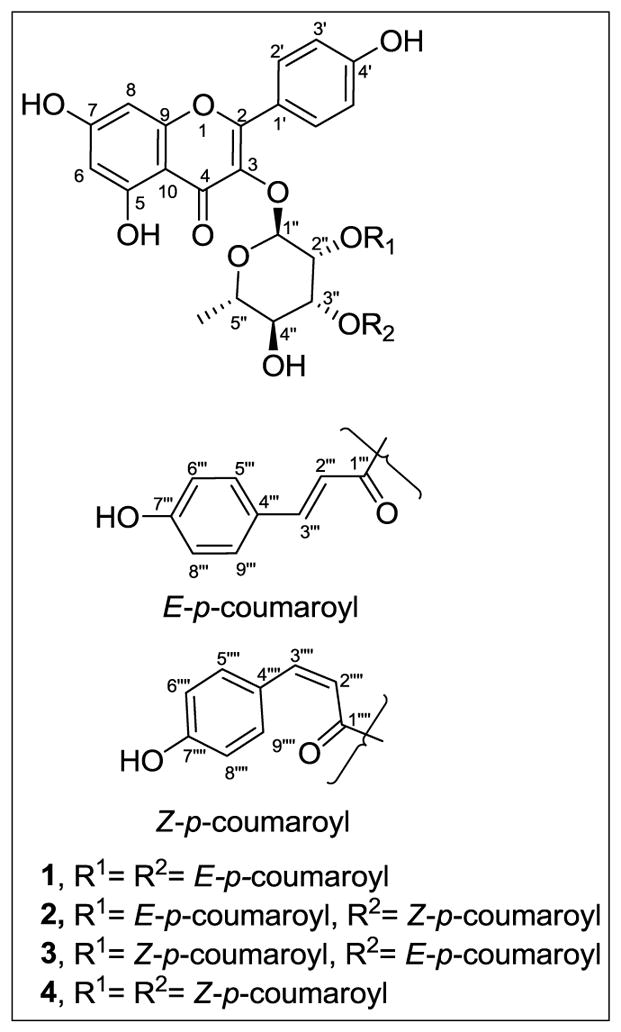
The structures of American sycamore glycosides **1**–**4**.

The discovery of novel, environmentally safe, natural antibacterial compounds would benefit aquaculturists due to the current limitations or the absence of efficacious management approaches available to producers for controlling the bacterial species responsible for columnaris disease, ESC, MAS, and streptococcosis. As part of our discovery process to identify active compounds against isolates of *F. columnare*, *E. ictaluri*, *A. hydrophila*, and *S. iniae*, natural compounds (Figure 1) from the American sycamore (*Plantus occidentalis* L. family Platanaceae) previously identified as antibacterial against methicillin-resistant *Staphylococcus aureus* (MRSA) [13] were evaluated in a rapid bioassay.

## Materials and Methods

### Test compounds

Four isolated compounds obtained from the leaves of the common American sycamore (*P. occidentalis*) were provided by Ironstone Separations, Inc., Etta, MS, USA. These four compounds were previously identified [13] as the following: kaempferol 3-*O*-*α*- L-(2*″*,3*″*-di-*E*-*p*-coumaroyl)rhamnoside (**1**), kaempferol 3-*O*-*α*-L- (2*″*-*E*-*p*-coumaroyl-3*″*-*Z*-*p*-coumaroyl)rhamnoside (**2**), kaempferol 3-*O*-*α*-L-(2*″*-*Z*-*p*-coumaroyl-3*″*-*E*-*p*-coumaroyl)rhamnoside (**3**), and kaempferol 3-*O*-*α*-L-(2*″*,3*″*-di-*Z*-*p*-coumaroyl)rhamnoside (**4**). A test mixture (**Mix**) of all four isomers (approximately 33% **1**, 23% **2**, 26% **3**, and 19% **4**) was also evaluated. The purity of each of the four test compounds and **Mix** was >95%.

### Microorganisms and preparation of culture material for bioassay

An isolate of *F. columnare* [isolate ALM-00-173 (genomovar II)] was obtained from Dr. Covadonga Arias (Department of Fisheries and Allied Aquacultures, Auburn University, Auburn, AL, USA). Cultures of *F. columnare* ALM-00-173 were maintained separately on modified Shieh (MS) agar plates (pH 7.2–7.4) [14] in order to assure purity. Prior to conducting the bioassay, single colonies of *F. columnare* ALM-00-173 were utilized in the preparation of the assay culture material by culturing in 75 mL of MS broth for 24 h at 29±1 °C at 150 rpm on a rotary shaker (model C24KC; New Brunswick Scientific, Edison, NJ, USA). After overnight incubation, a 0.5 McFarland standard of *F. columnare* ALM-00-173 culture material was prepared by transferring cells from the broth culture to fresh MS broth [15].

An isolate of *E. ictaluri* (isolate S02-1039) was obtained from Mr. Tim Santucci (formerly with the College of Veterinary Medicine, Mississippi State University, Stoneville, MS, USA). Cultures of *E. ictaluri* were maintained on 3.8% Mueller-Hinton (MH) agar plates (pH 7.3) (Becton, Dickinson and Company, Sparks, Maryland) in order to assure purity. Prior to conducting the bioassay, single colonies of *E. ictaluri* S02-1039 were utilized in the preparation of the assay culture material by aseptically transferring bacterial cells from colonies to 45 mL of 3.8% MH broth to form a bacterial cell density of 0.5 McFarland standard.

An isolate of *A. hydrophila* (isolate HSB-B2) was provided by Dr. Julie Bebak (formerly with the U.S. Department of Agriculture, Agricultural Research Service, Aquatic Animal Health Research Laboratory, Auburn, AL, USA), and this isolate was obtained from sunshine bass that had died during a farm outbreak. To ensure culture purity, *A. hydrophila* HSB-B2 was streaked on MH agar plates. Prior to conducting the bioassay, single colonies of *A. hydrophila* HSB-B2 were used to prepare the assay culture material by culturing *A. hydrophila* HSB-B2 in 50 mL of 3.8% MH broth at 29 ± 1°C at 150 rpm on a rotary shaker for 18 h in order to prepare the 0.5 MacFarland standard.

A culture of *S. iniae* (isolate LA94-426) was provided by Dr. Ahmed Darwish (formerly with the U.S. Department of Agriculture, Agricultural Research Service, Harry K. Dupree Stuttgart National Aquaculture Research Center, Stuttgart, AR, USA). In order to assure purity, cultures of *S. iniae* LA94-426 were maintained on plates of Columbia CNA agar containing 5% sheep blood (Remel, Inc., Lenexa, KS, USA). The bioassay culture material of *S. iniae* LA94-426 was prepared in the same manner as used for *F. columnare* ALM-00-173, except 3.8% MH broth was used and the broth cultures were incubated for 18 h prior to preparing the 0.5 MacFarland standard.

### Antibacterial bioassay

The test compounds were evaluated for antibacterial activity using a rapid 96-well microplate bioassay and following previous procedures [15]. Florfenicol and oxytetracycline HCl were included as positive drug controls. Control wells (no test material added) were also included in each assay. Test compounds and drug controls were dissolved separately in technical grade 100% ethanol. Final concentrations of test compounds and drug controls were 0.01, 0.1, 1.0, 10.0, and 100.0 μM. Three replications were used for each dilution of each test compound and controls. Final results were converted to units of mg/L to allow comparison with previous studies.

The 24-h 50% inhibition concentration (IC_50_) and minimum inhibitory concentration (MIC) were determined using sterile 96-well polystyrene microplates (Corning Costar Corp., Acton, Massachusetts) with flat-bottom wells. Initially, dissolved test compounds or drug controls were micropippeted separately into individual microplate wells (10 μL/well), and the ethanol was allowed to completely evaporate before 0.5 MacFarland bacterial culture was added to the microplate wells (200 μL/well). Microplates were incubated at 29±1°C (VWR model 2005 incubator; Sheldon Manufacturing, Inc., Cornelius, OR, USA). A Packard model SpectraCount microplate photometer (Packard Instrument Company, Meriden, CT, USA) was used to measure the absorbance (630 nm) of the microplate wells at time 0 and 24-h.

The means and standard deviations of absorbance measurements were calculated, graphed, and compared to controls to determine the 24-h IC_50_ and MIC for each test compound. The 24-h IC_50_ and MIC results for each test compound were divided by the respective 24-h IC_50_ and MIC results obtained for the positive controls florfenicol and oxytetracycline to determine the relative-to-drug-control florfenicol (RDCF) and relative-to-drug-control oxytetracycline (RDCO) values.

## Results and Discussion

Among the four isomers of the sycamore-derived antibacterial metabolite, **3** and **4** were more antibacterial against *F. columnare* ALM-00-173 based on 24-h IC_50_ values of 2.13±0.11 mg/L and 2.62±0.23 mg/L, respectively (Table 1). The mixture of all four isomers (**Mix**) also had similar activity, with a 24-h IC_50_ of 3.1±0.49 mg/L. The RDCF and RDCO 24-h IC_50_ values also indicated the greatest activities of **3**, **4**, and **Mix** against *F. columnare* ALM-00-173, with RDCO values closer to “1.0” for **3** and **4** which indicates similar antibacterial activities of these isomers to oxytetracycline. Essentially, all four isomers and **Mix** had the same or similar MIC values (7.47 mg/L) when considering the standard error of the mean for **3**. The RDCF and RDCO MIC values indicate substantially weaker antibacterial activity for three isomers (**1**, **2** and **4**) and **Mix** compared to the two drug controls while **3** had approximately one order of magnitude less antibacterial activity than the drug controls.

Evaluation of the four isomers and mixture of the isomers against *E. ictaluri* S02-1039 revealed no activity at the concentrations tested (Table 2). In addition, the four isomers and the mixture were also not antibacterial towards *A. hydrophila* HSB-B2 at test concentrations (Table 3). Therefore, the results demonstrate that these isomers do not possess broad-spectrum antibacterial activity towards species of Gram-negative, rod-shaped bacteria.

Based on 24-h IC_50_ results, **Mix** was slightly more antibacterial than any of the other individual four isomers against *S. iniae* LA94-426, with a value of 1.76±0.04 mg/L (Table 4). The 24-h IC_50_ RDCF values indicate that the four isomers and **Mix** were only slightly less active against *S. iniae* LA94-426 than florfenicol, with values slightly above “1” and **Mix** with the lowest value at 1.2±0. Conversely, the four isomers and **Mix** had 24-h IC_50_ RDCO values more than an order of magnitude higher than the RDCF values which indicates substantially less activity than the drug control oxytetracycline. The MIC results show similar activities among the four isomers and **Mix** against *S. iniae* LA94-426, with a range of 4.11 ± 3.36 to 7.47±0 mg/L. The MIC RDCF values indicate **3**, **4**, and **Mix** to be slightly more antibacterial than **1** and **2,** but all four isomers and **Mix** were substantially less active than the drug controls.

The previous report on the antibacterial activities of the four isolated kaempferol isomers found them to be relatively inactive against the Gram-negative bacteria *Escherichia coli* and *Pseudomonas aeruginosa* [13]. However, the current results are the first to report of their antibacterial activities against the Gram-negative bacterium *F. columnare*. Also, this is the first report on their antibacterial activities against *S. iniae*. A similar range of antibacterial activities (IC_50_ of 0.4 to 2.0 mg/L) for **1**, **2**, **3**, and **4** against MRSA, which is also a Gram-positive, coccus, has been previously reported [13]. In addition, **4** was the most antibacterial among the four isomers against MRSA (IC_50_ 0.4 mg/L) which is similar to our results for *S. iniae* LA94-426 (Table 4). Apparently, the *Z* configuration around the double bonds of the *p*-coumaroyl units as compared to the *E* configuration plays an important role in the enhancement of the antibacterial activity of these compounds [13].

The bioassay evaluation of such natural compounds is the first step in the discovery of novel natural compounds to potentially be used in the management of columnaris disease and streptococcosis. Future studies of the four isomers include challenge studies in order to more fully evaluate the effectiveness of these compounds for disease management.

## Figures and Tables

**Table 1:** Results of the bioassay evaluation of antimicrobial compounds from American sycamore leaves for antibacterial activity against *Flavobacterium columnare* ALM-00-173. Numbers in parentheses are the standard error of the mean.

			24-h IC_50_ a		MIC^b^	
Test compound	24-h IC_50_ a	MIC^b^	RDCF^c^	RDCO^d^	24-h IC_50_ c	MIC^d^
Flor*	0.58 (0.04)	0.36 (0)				
Oxy**	0.86 (0.07)	0.46 (0)				
**1**	17.18 (0.75)	7.47 (0)	29.9 (0.5)	20.3 (2.4)	20.8 (0)	16.2 (0)
**2**	6.91 (3.55)	7.47 (0)	12.4 (6.9)	7.8 (3.6)	20.8 (0)	16.2 (0)
**3**	2.13 (0.11)	4.11 (3.36)	3.7 (0)	2.5 (0.3)	11.4 (9.3)	8.9 (7.3)
**4**	2.62 (0.23)	7.47 (0)	4.6 (0.1)	3.1 (0.5)	20.8 (0)	16.2 (0)
**Mix**	3.1 (0.49)	7.47 (0)	5.5 (1.2)	3.6 (0.3)	20.8 (0)	16.2 (0)

* Florfenicol;

** Oxytetracyline HCl

a 24-h IC_50_ = 50% inhibition concentration in mg/L.

b MIC = Minimum inhibitory concentration in mg/L.

c RDCF = Relative-to-drug-control florfenicol; values closer to 1.0 indicate higher antibacterial activity compared to florfenicol.

d RDCO = Relative-to-drug-control oxytetracycline; values closer to 1.0 indicate higher antibacterial activity compared to oxytetracycline.

**Table 2:** Results of the bioassay evaluation of antimicrobial compounds from American sycamore leaves for antibacterial activity against *Edwardsiella ictaluri* S02-1039. Numbers in parentheses are the standard error of the mean.

			24-h IC_50_ a		MIC^b^	
Test compound	24-h IC_50_ a	MIC^b^	RDCF^c^	RDCO^d^	24-h IC_50_ c	MIC^d^
Flor*	0.21 (0.01)	0.36 (0)				
Oxy**	0.1 (0.01)	0.05 (0)				
**1**	>74.7	>74.7	>373.5	>747.0	>207.5	>149.4
**2**	>74.7	>74.7	>373.5	>747.0	>207.5	>149.4
**3**	>74.7	>74.7	>373.5	>747.0	>207.5	>149.4
**4**	>74.7	>74.7	>373.5	>747.0	>207.5	>149.4
**Mix**	>74.7	>74.7	>373.5	>747.0	>207.5	>149.4

* Florfenicol;

** Oxytetracyline HCl

a 24-h IC_50_ = 50% inhibition concentration in mg/L.

b MIC = Minimum inhibitory concentration in mg/L.

c RDCF = Relative-to-drug-control florfenicol; values closer to 1.0 indicate higher antibacterial activity compared to florfenicol.

d RDCO = Relative-to-drug-control oxytetracycline; values closer to 1.0 indicate higher antibacterial activity compared to oxytetracycline.

**Table 3:** Results of the bioassay evaluation of antimicrobial compounds from American sycamore leaves for antibacterial activity against *Aeromonas hydrophila* HSB-B2. Numbers in parentheses are the standard error of the mean.

			24-h IC_50_ a		MIC^b^	
Test compound	24-h IC_50_ a	MIC^b^	RDCF^c^	RDCO^d^	24-h IC_50_ c	MIC^d^
Flor*	0.18 (0.14)	0.36 (0)				
Oxy**	0.07 (0.01)	0.05 (0)				
**1**	>74.7	>74.7	>415.0	>1067.1	>207.5	>1494.0
**2**	>74.7	>74.7	>415.0	>1067.1	>207.5	>1494.0
**3**	>74.7	>74.7	>415.0	>1067.1	>207.5	>1494.0
**4**	>74.7	>74.7	>415.0	>1067.1	>207.5	>1494.0
**Mix**	>74.7	>74.7	>415.0	>1067.1	>207.5	>1494.0

* Florfenicol;

** Oxytetracyline HCl

a 24-h IC_50_ = 50% inhibition concentration in mg/L.

b MIC = Minimum inhibitory concentration in mg/L.

c RDCF = Relative-to-drug-control florfenicol; values closer to 1.0 indicate higher antibacterial activity compared to florfenicol.

d RDCO = Relative-to-drug-control oxytetracycline; values closer to 1.0 indicate higher antibacterial activity compared to oxytetracycline.

**Table 4:** Results of the bioassay evaluation of antimicrobial compounds from American sycamore leaves for antibacterial activity against *Streptococcus iniae* LA94-426. Numbers in parentheses are the standard error of the mean

			24-h IC_50_ a		MIC^b^	
Test compound	24-h IC_50_ a	MIC^b^	RDCF^c^	RDCO^d^	RDCF^c^	RDCO_d_
Flor*	1.49 (0.06)	0.36 (0)				
Oxy**	0.12 (0.02)	0.46 (0)				
**1**	2.24 (0.37)	7.47 (0)	1.5 (0.3)	19.7 (6.4)	20.8 (0)	16.2 (0)
**2**	2.1 (0.08)	7.47 (0)	1.4 (0)	17.9 (2.4)	20.8 (0)	16.2 (0)
**3**	2.1 (0.08)	4.11 (3.36)	1.4 (0)	17.9 (2.4)	11.5 (9.4)	8.9 (7.3)
**4**	1.87 (0.23)	4.11 (3.36)	1.3 (0.2)	16.3 (4.6)	11.5 (9.4)	8.9 (7.3)
**Mix**	1.76 (0.04)	4.11 (3.36)	1.2 (0)	15.0 (2.2)	11.5 (9.4)	8.9 (7.3)

* Florfenicol;

** Oxytetracyline HCl

a 24-h IC_50_ = 50% inhibition concentration in mg/L.

b MIC = Minimum inhibitory concentration in mg/L.

c RDCF = Relative-to-drug-control florfenicol; values closer to 1.0 indicate higher antibacterial activity compared to florfenicol.

d RDCO = Relative-to-drug-control oxytetracycline; values closer to 1.0 indicate higher antibacterial activity compared to oxytetracycline.
